# Histopathologic Differentiation between Enchondroma and Well-differentiated Chondrosarcoma: Evaluating the Efficacy of Diagnostic Histologic Structures

**DOI:** 10.5681/joddd.2011.022

**Published:** 2011-09-05

**Authors:** Shams Shariat Torbaghan, Mahdi Ashouri, Noushin Jalayer Naderi, Nima Baherini

**Affiliations:** ^1^Professor, Department of Pathology, Institute of Cancer, Faculty of Dentistry, Shahed University, Tehran, Iran; ^2^Oral and Maxillofacial Pathologist, Private Practice, Tehran, Iran; ^3^Assistant Professor, Department of Oral and Maxillofacial Pathology, Faculty of Dentistry, Shahed University, Tehran, Iran; ^4^Dentist, Private Practice, Tehran, Iran

**Keywords:** Enchondroma, Chondrosarcoma, Histopathologic differentiation, Clinical follow-up

## Abstract

**Background and aims:**

Well-differentiated chondrosarcoma and enchondroma are similar in histopathologic aspects; therefore, special methods should be used to make a distinction between these benign and malignant tumors. The aim of this study was to evaluate the efficacy of a histopathologic method in the long-term follow-up for differentiating these lesions.

**Materials and methods:**

The medical records of patients with histopathologic diagnosis of chondrosarcoma and en-chondroma were retrieved from the Institute of Cancer, Department of Pathology from 1981 to 2007 in this retrospective study. A total of 14 patients with chondrosarcoma and 7 patients with enchondroma were included. Tumor lobulation pat-tern and fibrous tissue around the lesions were used for histopathologic differentiation between well-differentiated chon-drosarcoma and enchondroma. Method accuracy was evaluated by tumor recurrence in the long-term follow-up.

**Results:**

In the well-differentiated chondrosarcoma group, the follow-up period was 97 months. All the patients (100%) experienced recurrence. In the enchondroma group, the follow-up period was 129 months. There was no recurrence in this group.

**Conclusion:**

Lobulation pattern and fibrous tissue formation around the tumor can be an effective and helpful indicator for histopathologic differentiation between enchondroma and well-differentiated chondrosarcoma.

## Introduction

Enchondroma is a benign chondroid tumor which consists of mature hyaline cartilage. Most of the tumors have limited growth potential. Asymptomatic small lesions do not need any treatment. Well-differentiated chondrosarcoma is a slow-growing malignant cartilaginous tumor with locally invasive and high recurrence potential. Radical surgical excision is the most effective mode of therapy. Both lesions contain mature hyaline cartilage with a lobular pattern and small chondrocytes in lacunar spaces.^1^ In well-differentiated chondrosarcoma, lacunae containing chondroid matrix are prominent features. This feature, in most instances, is very similar to enchondroma.^2^

Well-differentiated chondrosarcoma and enchondroma are very similar in histopathologic aspects. Since microscopic differentiation between these tumors is difficult, wrong diagnosis leads to wrong treatment plans. Different methods have been studied for differential diagnosis of well-differentiated chondrosarcoma from enchondroma.^3 - 6^ Although these methods are useful in the differential diagnosis, histologic study is a more superior process.

Based on histopathologic criteria, it is believed that presence of binucleated chondrocytes is required for the diagnosis of well-differentiated chondrosarcoma.^2^ In most studies, this definition has been used to make a distinction between well-differentiated chondrosarcoma and enchondroma.^7 - 9^

Loubular pattern is a defined histopathologic feature of well-differentiated chondrosarcoma; the lobules are separated by a fibrous tissue.^2^ Enchondroma lobules are regular and the fibrous tissue consists of mature connective tissue. On the other hand, well-differentiated chondrosarcoma consists of irregular lobules with cellular fibrous tissue around the tumor.^1 - 2^

Cytology is not sufficient to distinguish these two tumors. The results of studies about the presence of binucleated chondrocytes are not satisfactory.

In this study, fibrotic connective tissue formation and lobulation pattern around the tumor were used for differential diagnosis. Based on our knowledge, this is the first report about the fibrotic connective tissue formation and lobulation pattern for distinguishing enchondroma from well-differentiated chondrosarcoma. The aim of this study was to determine the efficacy of using fibrotic connective tissue formation and lobulation pattern around enchondroma and well-differentiated chondrosarcoma in a long-term follow-up to distinguish these two tumors.

## Materials and Methods

**Figure 1:**
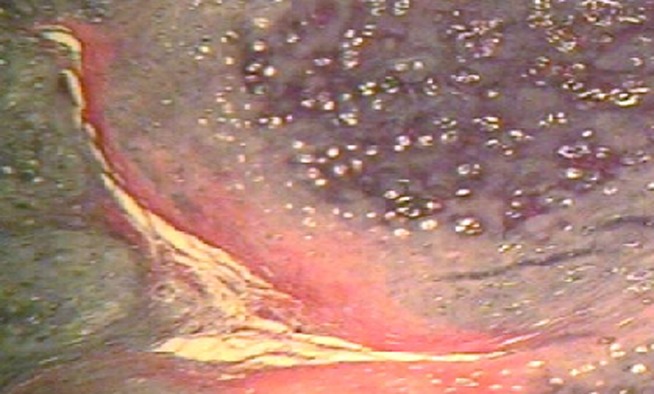
The lobulation pattern and fibrous tissue formation in enchondroma; the lobules are regular and the fibrous tissue is mature with low cellularity.

**Figure 2:**
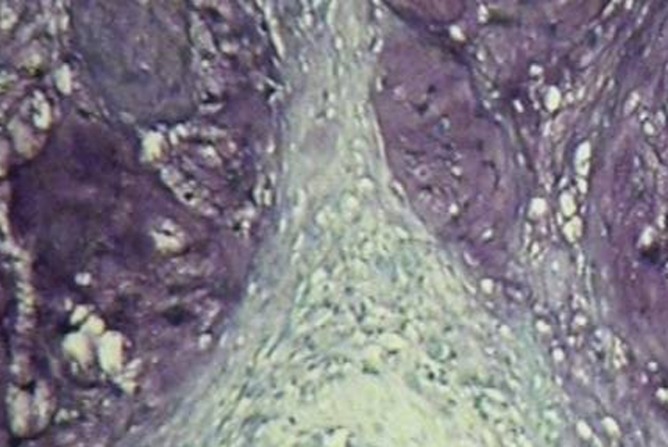
The lobulation pattern and fibrous tissue formation in well-differentiated chondrosarcoma; the lobules are irregular and the fibrous tissue is cellular with active blasts and many blood vessels.

The medical records of patients with histopathologic diagnosis of chondrosarcoma and enchondroma were retrieved from the Institute of Cancer, Department of Pathology, from 1981 to 2007. The medical records of all the patients with enchondroma and chondrosarcoma were included in the study. The incomplete information of records was the exclusion criteria. Patients’ data including age, gender, anatomic location, genetic background and clinical symptoms of the tumors were registered. The histopathologic sections of all the samples were examined again. Two histologic patterns including tumor lobulation and fibrous tissue production around the lesions were used for making a distinction between well-differentiated chondrosarcoma and enchondroma. According to the histopathologic definition of the tumor, lobulation of enchondroma is regular and the lobules are the same size but well-differentiated chondrosarcoma is composed of different sizes of irregular lobules. Fibrous tissue formation around the tumor is the second most important pattern. The fibrous tissue around enchondroma consists of hyalinized mature connective tissue with few cellularity and little blood vessels. The tumor cells are inactive. In well-differentiated chondrosarcoma, the fibrous tissue around the tumor is cellular with active blasts and many blood vessels.^1 - 2^ Figures 1 and 2 show the histopathologic pattern of lobulation and fibrous tissue formation in enchondroma and well-differentiated chondrosarcoma, respectively. Method efficacy was evaluated by tumor recurrence in a long-term follow-up. All the patients were asked about the signs such as pain and recurrence. A total of 61 records were included in the study. Because of insufficient information of medical records, phone numbers or address changes and lack of patient cooperation 40 patients were missing. A signs chart including pain, inflammation, type of treatment and recurrence information were completed for each patient. Data were compared between the two groups. Non-recurrent group was considered the enchondroma patients.

## Results

###  Enchondroma Group

There were 7 patients in this group. Average follow-up period was 129 months. The age range was 11 to 56 with an average of 30.2 years. All the patients were male. None of patients had genetic background. None of patients had experienced recurrence.

### Chondrosarcoma Group

A total of 14 patients were included in this group. Average follow-up period was 97 months. The age range was 18 to 57 with an average of 35.7 years. There were 7 (%50) males and 7 (%50) females in the group with a male:female ratio of 1:1. 50% of the patients had genetic background. All (100%) the patients suffered from recurrence. Two patients had passed away.

Clinical signs and treatment plan of enchondroma and well-differentiated chondrosarcoma are summarized in Table 1.

**Table 1:** Clinical signs and treatment plan of enchondroma and well-differentiated chondrosarcoma

Lesion	Pain	Inflammation	Pain & Inflammation	S	S, R	S, R, C	C	Recurrence
Enchondroma )n=7)	3(%42.85)	5(%71.42)	1(%14.28)	7(%100)	0	0	0	0
Chondrosarcoma (n=14)	9(%64.28)	8(%57.14)	4(%28.57)	2(%14.28)	8(%57.14)	2(%14.28)	2(%14.28)	12(%85.71)

S: Surgery; R: Radiotherapy; C: Chemotherapy.

**Table 2:** Chart sign of patients with enchondroma

No.	Age	Gender	Anatomic site	Genetic background	Fellow-up time	Clinical sign
1	37	Male	Metacarpus	No	152	Swelling
2	20	Male	Finger( foot)	No	164	Swelling
3	18	Male	Pharanx	No	120	Slight pain
4	11	Male	Arm	No	132	Swelling
5	16	Male	Finger(hand)	No	120	Swelling, Pain
6	56	Male	Arm	No	107	Swelling
7	54	Male	Scapula	No	124	Slight pain

**Table 3:** Chart sign of patients with well-differentiated chondrosarcoma

No.	Age	Gender	Anatomic site	Inheritance background	Fellow-up time (Months)	Clinical sign
1	36	Male	Pelvis	No	92	Swelling
2	25	Female	Pelvis	No	51	Swelling
3	56	Female	Leg	Yes	106	Swelling, Pain
4	21	Female	Pelvis	Yes	79	Swelling, Pain
5	57	Male	Tarsus	Yes	123	Swelling, Pain
6	42	Female	Pelvis	No	99	Swelling
7	36	Female	Pelvis	Yes	98	Swelling, Pain
8	38	Male	Leg	No	174	Swelling
9	34	Male	Sacral	No	49	Pain
10	57	Female	Shoulder	Yes	36	Pain
11	23	Male	Leg	No	103	Pain
12	18	Male	Loin	Yes	105	Pain
13	20	Female	Pelvis	No	72	Pain
14	28	Male	Pelvis	No	96	Pain

There was no recurrence in the enchondroma group. All the chondrosarcoma group cases had experienced recurrence.

## Discussion

Histopathologic diagnosis between well-differentiated chondrosarcoma and enchondroma is very difficult. In this study, a method based on lobulation pattern and fibrous tissue formation around the tumor was used in the long-term follow-up. There were no signs of recurrence in the enchondroma group. On the other hand, patients with well-differentiated chondrosarcoma had suffered recurrence. Two deaths had been registered in this group. The results showed that these two factors are efficient in distinguishing enchondroma from well-differentiated chondrosarcoma.

It has been hypothesized that large-size chondrocytes with double or multiple nuclei and cellularity are useful tools for differentiating benign and malignant cartilaginous lesions.^7 - 8^

As a traditional definition, presence of binucleated cells and mitotic figures are important features for the diagnosis of a cartilage tumor. With this definition, something is mising: the biologic behavior of benignancy or malignancy.

In addition to these features, the biologic nature of tumors must be taken into account. Therefore, in the present study fibrotic connective tissue formation and lobulation pattern of the tumors were used. Enchondroma consists of regular lobules and mature connective tissue with low cellularity; well-differentiated chondrosarcoma is composed of irregular lobules with highly cellular fibrous tissue.

Mirra et al^9^ showed that tissue pattern can be used for making a distinction between enchondroma and chondrosarcoma of the bones. With these patterns enchondroma consists of hyaline cartilage nodules surrounded by lamellar bone. The pattern of chondrosarcoma comprises a cartilage that permeates into marrow. Fibrotic bands form between the lobules.

Mirra et al reported that lobular pattern of enchondroma is different from that of chondrosarcoma, which is consistent with the results of the present study. In addition to lobular pattern, they used the infiltration pattern of tumor tissue. Since mapping of the pattern of bone infiltration is not possible in small pieces of incisional biopsy, using the natural tissue structure which reflects the biologic manner of tumor is very important.

Eefting et al^10^ proposed a set of 5 cytologic and tissue architectural features, including high cellularity, presence of host bone entrapment, open chromatin, mucoid matrix quality, and age above 45, for optimal differentiation between enchondroma and central grade I chondrosarcoma. They concluded that mucoid matrix degeneration more than 20% and/or entrapment of bone were effective parameters with 95% sensitivity and specificity for distinguishing enchondroma from central grade I chondrosarcoma.

The high cellularity of grade I chondrosarcoma versus enchondroma is in consistent with the results of the present study. Based on the results of the present study, lobulation pattern and fibrous tissue formation around the tumor are useful indicators for differentiation between enchondroma and well-differentiated chondrosarcoma; by considering the nuclear pattern, they can be effective markers for distinguishing enchondroma from well-differentiated chondrosarcoma.

Although histopathologic diagnosis is critical in distinguishing benign and malignant chondroid tumors, it must be kept in mind that clinical and radiographic aspects are very important guides for decision-making.

We concluded that enchondroma is a chondroid tumor with slow growth pattern and mild biologic course; therefore, it must have a regular lobulation structure. Fibrous capsule of enchondroma is almost inactive with little blood vessels and low cellularity. On the other hand, because of fast growth, chondrosarcoma produces an irregular, asymmetrical lobulation pattern. The blood vessels and active blast cells are more numerous in the chondrosarcoma capsule.

## Conclusion

Lobulation pattern and fibrous tissue formation around the tumor can be an effective and helpful indicator for histopathologic differentiation between enchondroma and well-differentiated chondrosarcoma. These tumors can be ditinguished by using tissue patterns derived from biologic nature.
